# A Predictive HQSAR Model for a Series of Tricycle Core Containing MMP-12 Inhibitors with Dibenzofuran Ring

**DOI:** 10.1155/2014/630807

**Published:** 2014-12-07

**Authors:** Jamal Shamsara, Ahmad Shahir-Sadr

**Affiliations:** ^1^Pharmaceutical Research Center, School of Pharmacy, Mashhad University of Medical Sciences, Mashhad 91775-1365, Iran; ^2^Molecular and Cellular Biology Research Center, School of Medicine, Sabzevar University of Medical Sciences, Sabzevar 96138-73136, Iran

## Abstract

MMP-12 is a member of matrix metalloproteinases (MMPs) family involved in pathogenesis of some inflammatory based diseases. Design of selective matrix MMPs inhibitors is still challenging because of binding pocket similarities among MMPs family. We tried to generate a HQSAR (hologram quantitative structure activity relationship) model for a series of MMP-12 inhibitors. Compounds in the series of inhibitors with reported biological activity against MMP-12 were used to construct a predictive HQSAR model for their inhibitory activity against MMP-12. The HQSAR model had statistically excellent properties and possessed good predictive ability for test set compounds. The HQSAR model was obtained for the 26 training set compounds showing cross-validated *q* ^2^ value of 0.697 and conventional *r* ^2^ value of 0.986. The model was then externally validated using a test set of 9 compounds and the predicted values were in good agreement with the experimental results (*r*_pred_^2^ = 0.8733). Then, the external validity of the model was confirmed by Golbraikh-Tropsha and *r*_*m*_^2^ metrics. The color code analysis based on the obtained HQSAR model provided useful insights into the structural features of the training set for their bioactivity against MMP-12 and was useful for the design of some new not yet synthesized MMP-12 inhibitors.

## 1. Introduction

Matrix metalloproteinases (MMPs) family enzymes can degrade extracellular matrix components by their proteolytic activity which depends on catalytic zinc ion [1]. The main role of macrophage metalloelastase (MMP-12) is degradation of elastin. Furthermore, MMP-12 is an interesting therapeutic target overexpressed in inflammatory pathological conditions (such as respiratory system diseases including asthma and chronic obstructive pulmonary disorder (COPD)) [2]. Effectiveness of MMP-12 inhibitors in reducing inflammation in respiratory system has been shown [3, 4].

The active site is highly conserved among MMPs with the exception of a loop region called S1′. S1′ pocket in MMPs active sites varies slightly among MMPs in both sequence and structure [5]. Despite available structural information, still the lack of selectivity remains as a main challenge for successfulness of MMPs inhibitors in clinical trials. Furthermore, intrinsic flexibility of MMPs active sites makes MMPs active site analysis more complicated [6, 7]. Therefore, in this study, a ligand based approach was used to modify the side chain in a series of MMP-12 inhibitors. HQSAR (hologram quantitative structure activity relationship) is a method for QSAR (quantitative structure activity relationship) studies whose reliability has been established [8]. In the present study, a HQSAR study on a series of tricycle cores containing MMP-12 inhibitors was carried out.

## 2. Methods

### 2.1. Obtaining Biological Data and Generation of Molecular Structures

**Figure 1:**
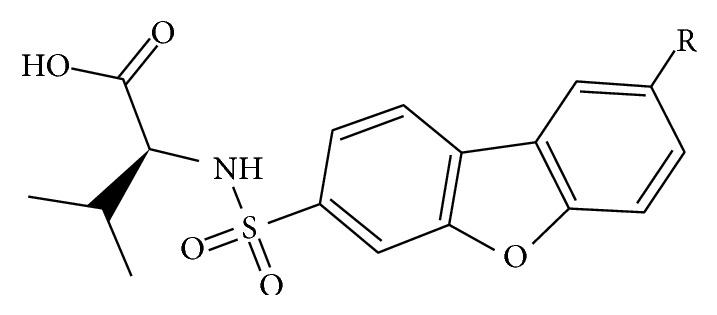
General structure for dataset.

The structures of 35 MMP-12 inhibitors and their biological activities for inhibition of MMP-12 were taken from the literatures (Figure 1 and Table 1) [9, 10]. As the activity of compound 10 is determined in both studies, we normalized the IC_50_ based on the reported activity for compound 10. The range of pIC_50_ (*µ*M) values for MMP-12 spans around three orders of magnitude (min = 1.6383, max = 4) in training set. The compounds were divided into two sets, training (*n* = 26) and test (*n* = 9) sets, according to the maintaining of structural diversity and the uniform distribution of IC_50_. The pIC_50_ (−Log IC_50_) was employed as dependent variable instead of IC_50_. The molecular structures were built using PyMOL (http://www.pymol.org/, The PyMOL Molecular Graphics System, Version 1.2r3pre, Schrödinger, LLC). The HQSAR model was developed by SYBYL-X1.2 molecular modeling package (Tripos International, St. Louis).

### 2.2. HQSAR Model Generation and Validation

HQSAR technique explores the contribution of each fragment of each molecule under study to the biological activity. As inputs, it needs datasets with their corresponding inhibitory activity in terms of pIC_50_. Structures in the dataset were fragmented and hashed into array bins. Molecular hologram fingerprints were then generated. Hologram was constructed by cutting the fingerprint into strings at various hologram length parameters.

After generation of descriptors, partial least square (PLS) methodology was used to find the possible correlation between dependent variable (−pIC_50_) and independent variable (descriptors generated by HQSAR structural features). LOO (leave-one-out) cross-validation method was used to determine the predictive value of the model. Optimum number of components was found out using results from LOO calculations. At this step, *q* ^2^ and standard error obtained from leave-one-out cross-validation roughly estimate the predictive ability of the model. This cross-validated analysis was followed by a non-cross-validated analysis with the calculated optimum number of principle components. Conventional correlation coefficient *r* ^2^ and standard error of estimate (SEE) indicated the validity of the model. The internal validity of the model was also tested by *Y*-randomization method [11]. In this test, the dependent variables are randomly shuffled while the independent variables (descriptors) are kept unchanged. It is expected that *q* ^2^ and *r* ^2^ calculated for these random datasets will be low. Finally, a set of compounds (which were not present in model development process) with available observed activity were used for external validation of the generated model. Predictive *r* ^2^ (*r*_pred_^2^) value was calculated using

(1) $$\begin{array}{c} r_{\text{pred}}^{2}=1-\frac{\text{PRESS}}{\text{SD}}; \end{array}$$

 PRESS: sum of the squared deviation between predicted and actual pIC_50_ for the test set compounds;
 SD: sum of the squared deviation between the actual pIC_50_ values of the compounds from the test set and the mean pIC_50_ value of the training set compounds.

The external validity of the model was also evaluated by Golbraikh-Tropsha [12] method and *r*_*m*_^2^ [13] metrics. For an acceptable QSAR model, the value of “average *r*_*m*_^2^” should be >0.5 and “delta *r*_*m*_^2^” should be <0.2. The applicability domain of the generated model was evaluated for both test and prediction sets by Euclidean based method. It calculates a normalized mean distance score for each compound in training set in range of 0 (least diverse) to 1 (most diverse). Then, it calculates the normalized mean distance score for compounds in an external set. If a score is outside the 0 to 1 range, it will be considered outside of the applicability domain. The external validity tests (Golbraikh-Tropsha and Rm^2^) and applicability domain test were done using tools available at http://dtclab.webs.com/software-tools.

### 2.3. Prediction Set (Design of New Compounds)

The prediction set contained 5 new not yet synthesized compounds having unknown observed values of activity against MMP-12. They were designed based on the prediction ability of developed HQSAR model.

### 2.4. Molecular Docking

The molecular docking process was carried out employing Glide (Glide, version 5.7, Schrödinger, LLC, New York, NY, 2011) using default parameters. The protein (3F17) was prepared using Protein Preparation Wizard. Hydrogens were added, bond orders were assigned, overlapping hydrogens were corrected, missing side chains were added, and water molecules were removed. Finally, the protein structure was minimized by OPLS2005 force field. The prepared protein structure containing inhibitor molecule was used for active site definition (within 13 A from cocrystalized ligand). The 2D maps of ligands-receptor interactions were generated by ligand interaction diagram (Schrödinger molecular modeling suite).

## 3. Results

### 3.1. HQSAR Model Predictivity

**Figure 2:**
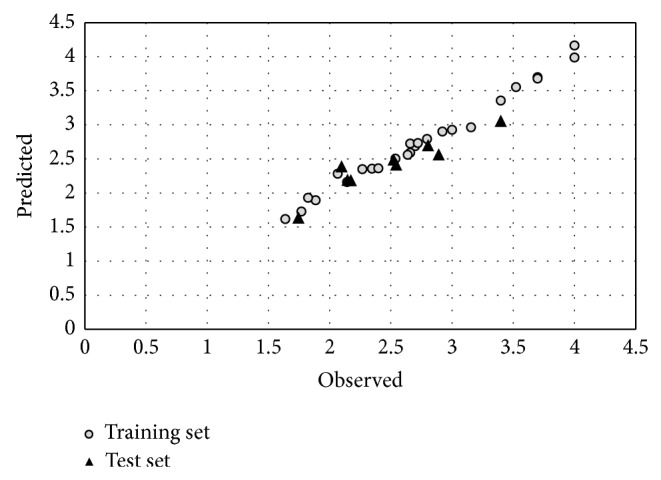
Plot of observed versus predicted activity obtained from HQSAR model for training and test sets.

The statistics for developed HQSAR model were shown in Table 2. The statistical parameters, *q* ^2^, *r* ^2^, SEE, and *r*_pred_^2^, showed the validity of our model. The best hologram model was generated using histogram length of 199 having six optimum components. Descriptors used for model generation were atoms, connections, and hydrogen atoms. The best generated model had cross-validated *q* ^2^ of 0.697 and non-cross-validated *r* ^2^ value of 0.986 with a standard error of 0.93. The total collection of the generated models for various histogram lengths comprises ensemble, and the ensemble value for *r* ^2^ was found to be 0.528. The *Y*-randomization results indicated that the calculated *q* ^2^ (−1.238, −0.303, −0.793, 0.081, and −0.146) and *r* ^2^ (0.683, 0.088, 0.086, 0.241, and 0.126) for five random models are very low which also confirm the internal validity of the generated HQSAR model. The results of *r*_pred_^2^ calculation showed that the proposed HQSAR model was reliable and could successfully predict pIC_50_ for structurally related compounds which were not included in development of the models. The *r*_*m*_^2^ (after scaling) was 0.825 and delta *r*_*m*_^2^ was 0.107. Additionally, all four conditions of The Golbraikh-Tropsha method are satisfied (*r* ^2^ = 0.876). Predicted values for the activity of molecules are shown in Table 1 and the experimental pIC_50_ against the values predicted by the HQSAR models are plotted (Figure 2).

### 3.2. HQSAR Atomic Contribution Plot

**Figure 3:**
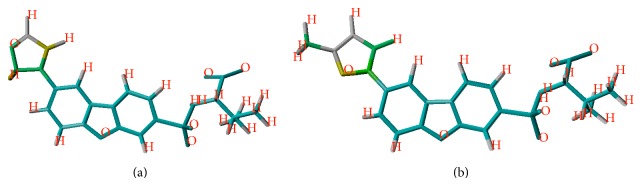
Contribution plot obtained from hologram quantitative structure activity relationship model for (a) compound 19 and (b) compound 20.

The generated model can be accessed through atomic contribution plot. The various colors of each atom correspond to various degrees of contribution towards the overall biological activity. Red, red orange, and orange depicted that the color belonging atoms were contributing negatively to the generated HQSAR model while colors reflecting yellow, green, and green blue were contributing positively to the model. Intermediate contributions were reflected by gray atom. The maximum common substructure was shown in cyan.  Figure 3 depicts the contribution of the most potent compound 20 as well as compound 19.

### 3.3. Prediction Set (Design of New Virtual Compounds)

This work allowed prediction of the activity of a set shown in Table 3 (not yet synthesized molecules). Their inhibitory activities were calculated according to the HQSAR model. They were designed based on compound 19 (one of the most active compounds). We had proposed a set of 5 new structures; some of them may show improved experimental MMP-12 inhibitory activity in comparison with the parent compound. This hypothesis and their selectivity should be verified experimentally.

### 3.4. Molecular Docking

**Figure 4:**
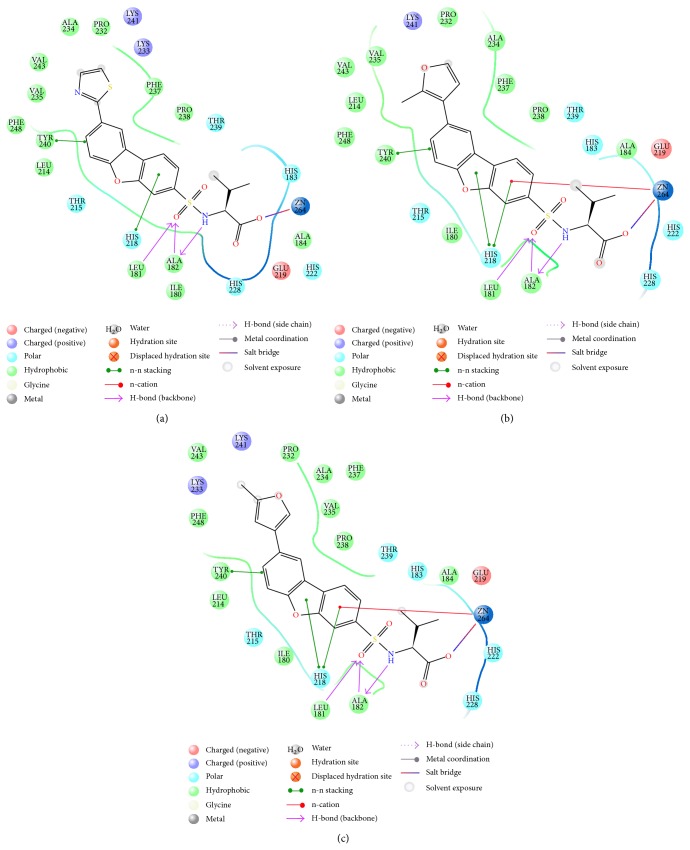
2D interaction diagram of 3 docked designed compounds: (a) structure 26, (b) structure 20, and (c) structure n3.

**Figure 5:**
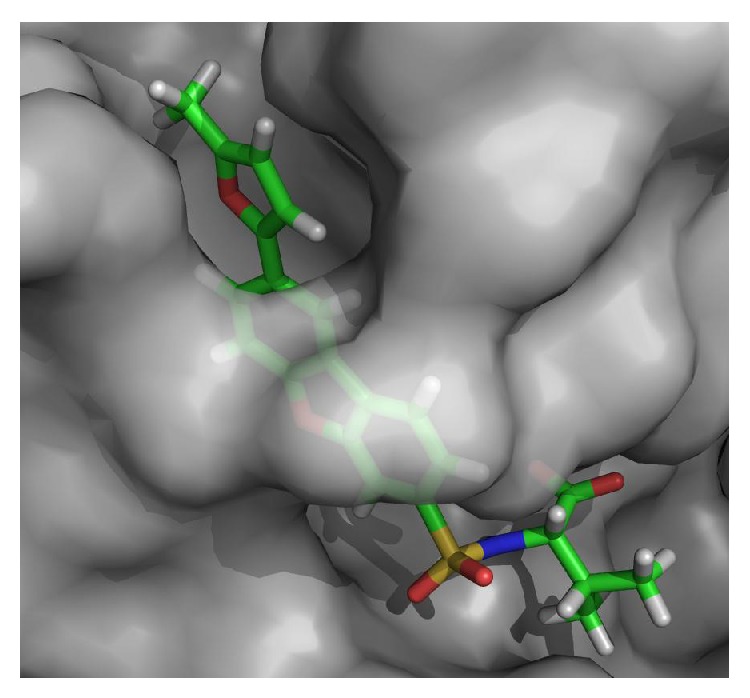
Compound 20 in the active site of MMP-12 (docked by Glide).

The molecular docking approach was employed to further analyze the ability of designed compounds in inhibition of MMP-12. In Table 3, the docking scores of the 3 new designed compounds and 3 molecules from train set were reported. The binding positions of all new compounds were inspected for their binding conformation and interactions in MMP-12 active site. For n3, 2D diagram of ligand-receptor interaction was presented (Figure 4(c)). The various heterocyclic rings substituted on the dibenzofuran scaffold do not seem to have strong interactions with the binding pocket of MMP-12 as it was suggested previously by X-ray crystallography [9]. However, if they have undesired properties they cannot fit in the narrow deep S1′ pocket of MMP-12. On the other hand, they can induce steric hindrance that prevents other parts of the molecule to have strong interactions with residues in the binding pocket. The conformation of the heterocyclic ring upon ligand binding is demonstrated in Figure 5.

## 4. Discussion

We successfully developed a HQSAR model for prediction of some MMP-12 inhibitors with good internal and external validity. Subsequently, the model was used to predict the activity of new MMP-12 inhibitors. The binding energy of new not yet synthesized molecules was evaluated by molecular docking.

Crystal structures have provided useful information for developing selective inhibitors toward particular MMPs including MMP-12. The segment 241–245 of MMPs (MMP-1 numbering) has the highest sequence variability among the various MMP enzymes and could be a target for designing selective inhibitors. However, this segment is very flexible which makes the molecular modeling predictions using 3D structures of MMPs inaccurate [14, 15]. Only some small differences in the sizes of hydrophobic side chains were seen. For example, Val 235 in MMP-12 is replaced by Leu214 in MMP-13 which makes the MMP-13 binding pocket smaller and more hydrophobic. The series of MMP-12 inhibitors employed in this study had carboxylic acid zinc binding group. Changing the R group that was placed in hydrophobic pocket of MMP-12 active site altered the potency and selectivity of these inhibitors. We modified this R group for fine tuning and designed new compound with promising MMP-12 inhibitory activity.

In the present study, we used HQSAR approach for a set of MMP-12 inhibitors. Contribution plot (Figure 3) showed that the green aromatic carbon was contributing positively to the model. Oxygen atom in furan ring was depicted by green or yellow and was contributing to the biological activity. Hydrogen molecules were rendered green, yellow, or white indicating that they showed intermediate contribution. The bulky group (methyl) was green indicating that it was contributing positively to the generated model, and it can be explained from the example that compound 20 was showing high potency. The developed model was used for the design of 5 new molecules. Overall docked conformation of the training set of inhibitors and new compounds in MMP-12 active site was similar to one determined by crystallography. Among new designed compounds, compounds n1, n2, and n3 had low SEP and their predicted activities were more reliable. Furthermore, compound n3 has the lowest docked energy.

## 5. Conclusion

In summary, we have developed a reliable HQSAR model for a series of tricycle cores containing MMP-12 inhibitors with dibenzofuran ring using activity data reported earlier [9, 10]. We used HQSAR analysis to design new not yet synthesized potent MMP-12 inhibitors. Their binding energies were evaluated by docking studies but for further validation it needs synthesize of the proposed new compounds and subsequent enzyme inhibition study.

## Figures and Tables

**Table 1:** Actual and predicted activities of the training and test sets based on the HQSAR model. Activities were shown as pIC_50_ (*μ*M).

Name	R	Actual pIC_50_ values	Predicted pIC_50_ values	Residues	Normalized mean distance score
10	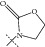	2.699	2.594	0.105	0.066

11	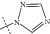	1.8861	2.05	−0.1639	0.028

12	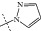	1.8239	2.144	−0.3201	0.022

13	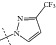	3.1549	2.688	0.4669	0.049

14	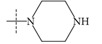	1.6383	1.646	−0.0077	0.332

15^a^	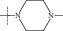	1.7447	1.754	−0.0093	0.065

16	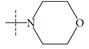	2.6576	2.672	−0.0144	0.208

19	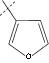	3.3979	3.706	−0.3081	0.037

20	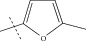	4	4.032	−0.032	0.043

21	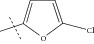	4	3.778	0.222	0.03

22	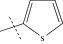	3.699	3.647	0.052	0.033

23	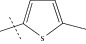	3.699	3.752	−0.053	0.031

24	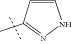	3	3.049	−0.049	0.005

25^a^	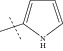	3.3979	3.17	0.2279	0.085

26	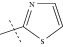	3	2.945	0.055	0.009

27	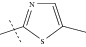	2.9208	2.949	−0.0282	0.008

33	Methyl	2.0655	2.341	−0.2755	0

34	Ethyl	2.5376	2.452	0.0856	0.01

35	i-Propyl	2.3468	2.423	−0.0762	0.087

36	t-Butyl	1.7696	1.839	−0.0694	0.554

37	i-Butyl	2.2676	2.203	0.0646	0.284

38	CH2OCH3	2.7212	2.571	0.1502	0.007

39	CF3	2.6576	2.543	0.1146	0

40	Cyclopropyl	2.7959	2.767	0.0289	0.08

41	Cyclobutyl	2.6383	2.689	−0.0507	0.377

42	Cyclohexyl	2.1427	2.126	0.0167	1

43	Phenyl	2.3979	2.561	−0.1631	0.116

44	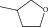	3.5229	3.491	0.0319	0.186

51^a^	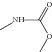	2.5441	2.483	0.0611	0.059

52^a^	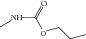	2.0969	2.502	−0.4051	0.088

53^a^	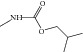	2.173	2.146	0.027	0.297

54^a^	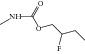	2.5229	2.526	−0.0031	0.049

55^a^	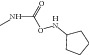	2.1461	2.305	−0.1589	0.324

56^a^	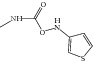	2.8909	2.616	0.2749	

57^a^	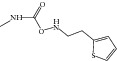	2.8037	2.773	0.0307	0.668

^a^Test set compounds.

**Table 2:** Statistical characteristics of the developed HQSAR model.

Parameter	Value
Number of compounds included in training set	26
Optimum number of components used in the PLS analysis	6
*q* ^2^ (cross-validated correlation coefficient)	0.697
SEE^a^ (*q* ^2^)	0.428
*r* ^2^ (non-cross-validated correlation coefficient)	0.986
SEE (*r* ^2^)	0.093
*r* ^2^ (ensemble^b^)	0.528
SEE (ensemble)	0.530
Best hologram length	199
Used information	Atoms + Connections + Hydrogen atoms
*r*_pred_^2^	0.8733

^a^SEE: standard error of estimate. ^b^For each hologram length, a model could be established. The collection of these models comprises the ensemble.

**Table 3:** Predicted activities for the molecules based on the HQSAR model. Activities were shown as pIC_50_ (*μ*M).

Name	R	Predicted pIC_50_ values	SE of prediction	Normalized mean distance score	Docking score
n1	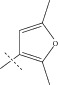	4.182	0.158579	0.161	−12.038972

n2	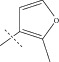	3.89	0.097741	0.076	−13.92

n3	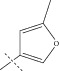	3.942	0.093502	0.072	−13.87

n4	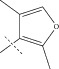	3.846	0.308627	0.291	—^a^

n5	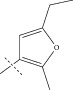	4.003	0.325568	0.315	—^a^

20 (observed = 4)	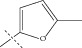	4.032	—	0.043	−13.7908

19 (observed = 3.3979)	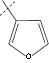	3.706	—	0.037	−12.555956

26 (observed = 3)	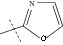	2.945	—	0.009	−11.781243

^a^Docking was not performed for these compounds.
